# Supplementary material for: Patterns of Intron Gain and Loss in Fungi
Source: PLoS Biol. 2004 Nov 30;2(12):e422. doi: 10.1371/journal.pbio.0020422 (PMC532390; doi:10.1371/journal.pbio.0020422)
Supplement: Table S1 — Also available at http://genes.mit.edu/NielsenEtAl/. (4.3 MB ZIP). [file pbio.0020422.st001.zip › NielsenEtAl/html/951.html]

AN5512.1.NCU07321.1.MG04948.1.FG04986.1


```
 CLUSTAL W (1.82) Multiple Sequence Alignments - Introns Inserted


Sequence 1: NCU07321.1	272 aa
Sequence 2: FG04986.1	225 aa
Sequence 3: AN5512.1	215 aa
Sequence 4: MG04948.1	263 aa
Alignment Length: 301 aa
Number Identitical Residues: 57 aa
Alignment Score (without introns) 3679


MG04948.1 	MAKKLSK-ANAAAAHSSVNTTHSDSTQTSNFSSSSSVNKAGLSAPGPSSLPPVLTDE-EP
NCU07321.1	MPRKLSKSRNVYAPASSSSS-----------SSSHSTTNLLTPYPQNDGLPPFLTDG-LP
FG04986.1 	MPRKLSK--NPLPPSSSTLS-----------SMSSASG-------ASSLLPSSLADPSLP
AN5512.1  	-MIRARR-TNTAVPIEDT-----------------------------SNAPSTFTDG-LP
          	   :  :  *   . ..                              .  *. ::*   *

MG04948.1 	LPRLIVFDLDYT0LWPFWVDTHVTPPIKAVAPADKSQHCSTVTDK~IGDTYSFYSDVPRM
NCU07321.1	LPSLVVLDLDYT~LWPFYSDCHPWPPLRALSG-------SVLSDR~NGENFSFYKHVPII
FG04986.1 	LPKLIVFDLDYT~LWPFWIDTHVTPPLKPNSSH------TSATDR~YGEDYGFFSDVPAI
AN5512.1  	LPKLIAFDLDYT~LWPFWVDTHVTPPIKARD------NNSRCTDK2WNESFAFYPHVNSI
          	** *:.:***** ****: * *  **::.          :  :*:  .: :.*: .*  :

MG04948.1 	LYTLPMAGVRLAVASRTHAPDLARDVLKQLQIPPYAAAG----GLSTLANNPNMPATPAA
NCU07321.1	LHLLQVAGVKLAVASKSPVGDLCREMLKLLRIPGGLPTSVEGFRRSPATTKGAAGAAADG
FG04986.1 	LYALPRAGIKIGVASRTSAPSLARDLLKMLHIT-----------------------APEG
AN5512.1  	LTSCKSRSIPLALASRTHAPDLARDMLKQLHIIP------------------NFSDNPSA
          	*      .: :.:**:: . .*.*::** *:*                         . .

MG04948.1 	TQKQ----------------------LAKEKPRRALDIFDAGLEIYPSSKVRHFDAIQKR
NCU07321.1	NGDGEGEEEDNSVKKTKTKGSGSGGGLTNEKGRKTIELFDGGLEIYEGTKLRHFEVIQKR
FG04986.1 	N--------------------------TKEKPKKALDMFDGLLEIYPGCKIKHFESLQKR
AN5512.1  	N---------------------------KARTVRALDYFDY-IQIFPATKTQHFSRIHQA
          	.                           : :  :::: **  ::*: . * :**. ::: 

MG04948.1 	TGIPYSEMIFFDDEARNKDVEQALGVLFCHVKDGMTWDELELGVRRWRRR---RQN----
NCU07321.1	TGIPYEEMLFFDDERPNLEVER-VGVTMQLIKDGLDWEELEKGIQKWRANRGFA------
FG04986.1 	TGIKYEDMLFFDDEARNRDTES-LGVTMCLVKDGVTWDELERGVTQWRNKRGYIKRPDAI
AN5512.1  	SGIAYEDMLFFDDEARNRNVETELGVTFLLVRDGMTRDEVDKGVWAWRRRNGIKQNQNQE
          	:** *.:*:*****  * :.*  :** :  ::**:  :*:: *:  ** . .  .  .  

MG04948.1 	---
NCU07321.1	---
FG04986.1 	ELE
AN5512.1  	G--
          	
```
